# Phenyl *N*-(4-methoxy­phen­yl)carbamate

**DOI:** 10.1107/S1600536809028785

**Published:** 2009-07-25

**Authors:** Zheng Fang, Yong-Lu Wang, Li-Li Ren, Ping Wei

**Affiliations:** a School of Pharmaceutical Sciences, Nanjing University of Technology, Nanjing 210009, People’s Republic of China; b School of Biotechnology and Pharmaceutical Engineering, Nanjing University of Technology, Nanjing 210009, People’s Republic of China

## Abstract

The asymmetric unit of the title compound, C_14_H_13_NO_3_, contains two crystallographically independent mol­ecules, in which the aromatic rings are oriented at dihedral angles of 75.64 (3) and 83.14 (3)°. An N—H⋯O hydrogen bond links the two mol­ecules. Weak intramolecular C—H⋯O inter­actions are observed in the two mol­ecules. In the crystal structure, inter­molecular N—H⋯O and C—H⋯O inter­actions link the mol­ecules into a two-dimensional network.

## Related literature

For bond-length data, see: Allen *et al.* (1987 ▶).
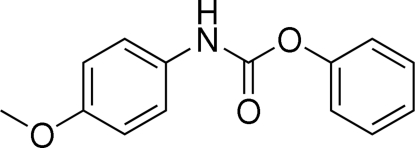


## Experimental

### 

#### Crystal data

C_14_H_13_NO_3_
*M* *_r_* = 243.25
Monoclinic, 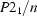

*a* = 9.869 (2) Å
*b* = 10.870 (2) Å
*c* = 23.319 (5) Å
β = 100.27 (3)°
*V* = 2461.5 (9) Å^3^
*Z* = 8
Mo *K*α radiation
μ = 0.09 mm^−1^
*T* = 294 K
0.30 × 0.20 × 0.10 mm

#### Data collection

Enraf–Nonius CAD-4 diffractometer
Absorption correction: ψ scan (North *et al.*, 1968 ▶) *T*_min_ = 0.973, *T*_max_ = 0.991
4733 measured reflections
4459 independent reflections
2109 reflections with *I* > 2σ(*I*)
*R*_int_ = 0.045
3 standard reflections frequency: 120 min intensity decay: 1%

#### Refinement

*R*[*F* ^2^ > 2σ(*F* ^2^)] = 0.069
*wR*(*F* ^2^) = 0.184
*S* = 1.03
4459 reflections
319 parameters
H-atom parameters constrained
Δρ_max_ = 0.43 e Å^−3^
Δρ_min_ = −0.34 e Å^−3^

### 

Data collection: *CAD-4 Software* (Enraf–Nonius, 1989 ▶); cell refinement: *CAD-4 Software*; data reduction: *XCAD4* (Harms & Wocadlo, 1995 ▶); program(s) used to solve structure: *SHELXS97* (Sheldrick, 2008 ▶); program(s) used to refine structure: *SHELXL97* (Sheldrick, 2008 ▶); molecular graphics: *ORTEP-3 for Windows* (Farrugia, 1997 ▶) and *PLATON* (Spek, 2009 ▶); software used to prepare material for publication: *SHELXL97*.

## Supplementary Material

Crystal structure: contains datablocks global, I. DOI: 10.1107/S1600536809028785/hk2744sup1.cif

Structure factors: contains datablocks I. DOI: 10.1107/S1600536809028785/hk2744Isup2.hkl

Additional supplementary materials:  crystallographic information; 3D view; checkCIF report

## Figures and Tables

**Table 1:** Hydrogen-bond geometry (Å, °)

*D*—H⋯*A*	*D*—H	H⋯*A*	*D*⋯*A*	*D*—H⋯*A*
N1—H1*A*⋯O5	0.86	2.19	3.038 (4)	170
N2—H2*B*⋯O2^i^	0.86	2.22	3.062 (4)	166
C6—H6*A*⋯O5^ii^	0.93	2.58	3.435 (5)	153
C9—H9*A*⋯O2	0.93	2.52	2.967 (5)	110
C23—H23*A*⋯O2^i^	0.93	2.60	3.390 (4)	144
C27—H27*A*⋯O5	0.93	2.30	2.907 (5)	122

Symmetry codes: (i) 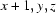; (ii) 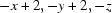.

**Fig. 1.**
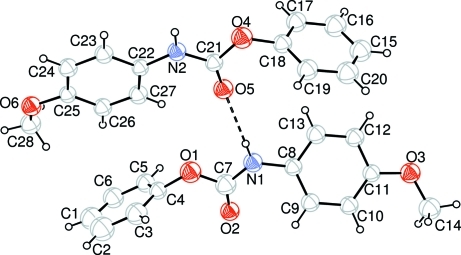
The molecular structure of the title molecule, with the atom-numbering scheme. Hydrogen bond is shown as dashed line.

**Fig. 2.**
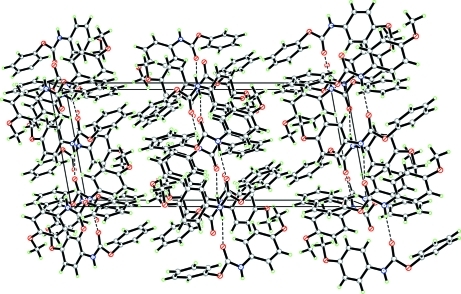
A partial packing diagram of the title compound. Hydrogen bonds are shown as dashed lines.

## supplementary crystallographic information

### Comment

Some derivatives of benzoic acid are important chemical materials. We report herein the crystal structure of the title compound.

The asymmetric unit of the title compound contains two crystallographically independent molecules (Fig. 1), in which the bond lengths (Allen *et al.*, 1987) and angles are within normal ranges. Rings A (C1-C6), B (C8-C13) and C (C15-C20), D (C22-C27) are, of course, planar and they are oriented at dihedral angles of A/B = 75.64 (3) and C/D = 83.14 (3)°. Intramolecular N-H···O hydrogen bond (Table 1) link the two molecules (Fig. 1). There also exist two intramolecular C-H···O interactions (Table 1).

In the crystal structure, intramolecular N-H···O and intermolecular N-H···O and C-H···O interactions (Table 1) link the molecules into a two dimensional network (Fig. 2), in which they may be effective in the stabilization of the structure.

### Experimental

For the preparation of the title compound, to a cold stirring solution of 4-methoxybenzenamine (1.0 g) and triethylamine (0.8 ml) in methylene chloride (10 ml) was added phenyl chloroformate (1.0 ml) slowly at 273 K. The mixture was then warmed and stirred for 1 h at room temperature. The mixture was washed with water (20 ml), dried over sodium sulfate, and concentrated to near dryness. The crude product was purified by recrystallization from petroleum ether (yield; 1.3 g). Crystals suitable for X-ray analysis were obtained by slow evaporation of a petroleum ether solution.

### Refinement

H atoms were positioned geometrically, with N-H = 0.86 Å (for NH) and C-H = 0.93 and 0.96 Å for aromatic and methyl H, respectively, and constrained to ride on their parent atoms, with U_iso_(H) = xU_eq_(C,N), where x = 1.5 for methyl H and x = 1.2 for all other H atoms.

### Crystal data

**Table:**

C_14_H_13_NO_3_	*F*(000) = 1024
*M**_r_* = 243.25	*D*_x_ = 1.313 Mg m^−^^3^
Monoclinic, *P*2_1_/*n*	Mo *K*α radiation, λ = 0.71073 Å
Hall symbol: -P 2yn	Cell parameters from 25 reflections
*a* = 9.869 (2) Å	θ = 10–13°
*b* = 10.870 (2) Å	µ = 0.09 mm^−^^1^
*c* = 23.319 (5) Å	*T* = 294 K
β = 100.27 (3)°	Block, colorless
*V* = 2461.5 (9) Å^3^	0.30 × 0.20 × 0.10 mm
*Z* = 8	

### Data collection

**Table:**

Enraf–Nonius CAD-4 diffractometer	2109 reflections with *I* > 2σ(*I*)
Radiation source: fine-focus sealed tube	*R*_int_ = 0.045
graphite	θ_max_ = 25.3°, θ_min_ = 1.8°
ω/2θ scans	*h* = 0→11
Absorption correction: ψ scan (North *et al.*, 1968)	*k* = 0→13
*T*_min_ = 0.973, *T*_max_ = 0.991	*l* = −28→27
4733 measured reflections	3 standard reflections every 120 min
4459 independent reflections	intensity decay: 1%

### Refinement

**Table:**

Refinement on *F*^2^	Primary atom site location: structure-invariant direct methods
Least-squares matrix: full	Secondary atom site location: difference Fourier map
*R*[*F*^2^ > 2σ(*F*^2^)] = 0.069	Hydrogen site location: inferred from neighbouring sites
*wR*(*F*^2^) = 0.184	H-atom parameters constrained
*S* = 1.03	*w* = 1/[σ^2^(*F*_o_^2^) + (0.08*P*)^2^] where *P* = (*F*_o_^2^ + 2*F*_c_^2^)/3
4459 reflections	(Δ/σ)_max_ < 0.001
319 parameters	Δρ_max_ = 0.43 e Å^−^^3^
0 restraints	Δρ_min_ = −0.34 e Å^−^^3^

### Special details

**Table:**

Geometry. All e.s.d.'s (except the e.s.d. in the dihedral angle between two l.s. planes) are estimated using the full covariance matrix. The cell e.s.d.'s are taken into account individually in the estimation of e.s.d.'s in distances, angles and torsion angles; correlations between e.s.d.'s in cell parameters are only used when they are defined by crystal symmetry. An approximate (isotropic) treatment of cell e.s.d.'s is used for estimating e.s.d.'s involving l.s. planes.
Refinement. Refinement of *F*^2^ against ALL reflections. The weighted *R*-factor *wR* and goodness of fit *S* are based on *F*^2^, conventional *R*-factors *R* are based on *F*, with *F* set to zero for negative *F*^2^. The threshold expression of *F*^2^ > σ(*F*^2^) is used only for calculating *R*-factors(gt) *etc*. and is not relevant to the choice of reflections for refinement. *R*-factors based on *F*^2^ are statistically about twice as large as those based on *F*, and *R*- factors based on ALL data will be even larger.

### Fractional atomic coordinates and isotropic or equivalent isotropic displacement parameters (Å^2^)

**Table:**

	*x*	*y*	*z*	*U*_iso_*/*U*_eq_	
O1	1.0212 (2)	0.7911 (3)	0.03978 (13)	0.0894 (8)	
O2	0.8011 (2)	0.7329 (2)	0.01287 (11)	0.0730 (8)	
O3	0.6882 (3)	0.3624 (3)	−0.20767 (12)	0.0811 (8)	
O4	1.4527 (2)	0.6417 (3)	−0.07369 (12)	0.0818 (9)	
O5	1.2693 (2)	0.7162 (2)	−0.03922 (11)	0.0731 (8)	
O6	1.4470 (2)	1.1216 (2)	0.16973 (11)	0.0725 (8)	
N1	0.9696 (3)	0.6635 (3)	−0.03360 (15)	0.0730 (9)	
H1A	1.0567	0.6696	−0.0331	0.088*	
N2	1.4897 (3)	0.7712 (3)	−0.00016 (13)	0.0618 (8)	
H2B	1.5736	0.7542	−0.0026	0.074*	
C1	0.9618 (5)	1.0279 (5)	0.1698 (3)	0.1066 (17)	
H1B	0.9538	1.0851	0.1986	0.128*	
C2	1.0165 (5)	0.9067 (6)	0.1852 (2)	0.1185 (18)	
H2A	1.0409	0.8824	0.2239	0.142*	
C3	1.0310 (4)	0.8289 (5)	0.1404 (2)	0.0975 (15)	
H3A	1.0668	0.7505	0.1486	0.117*	
C4	0.9940 (3)	0.8648 (4)	0.08460 (18)	0.0628 (10)	
C5	0.9390 (4)	0.9807 (4)	0.07198 (19)	0.0745 (11)	
H5A	0.9125	1.0061	0.0335	0.089*	
C6	0.9246 (4)	1.0556 (5)	0.1160 (2)	0.0919 (14)	
H6A	0.8851	1.1325	0.1069	0.110*	
C7	0.9180 (4)	0.7285 (4)	0.0059 (2)	0.0894 (8)	
C8	0.8940 (4)	0.5852 (3)	−0.07637 (18)	0.0628 (10)	
C9	0.7833 (4)	0.5173 (4)	−0.06612 (18)	0.0689 (11)	
H9A	0.7568	0.5211	−0.0298	0.083*	
C10	0.7113 (4)	0.4439 (4)	−0.10902 (17)	0.0684 (11)	
H10A	0.6362	0.3990	−0.1016	0.082*	
C11	0.7492 (4)	0.4364 (4)	−0.16274 (18)	0.0627 (10)	
C12	0.8617 (4)	0.5036 (4)	−0.17297 (18)	0.0724 (11)	
H12A	0.8891	0.4986	−0.2090	0.087*	
C13	0.9328 (4)	0.5771 (4)	−0.13035 (19)	0.0736 (11)	
H13A	1.0079	0.6221	−0.1378	0.088*	
C14	0.5858 (5)	0.2793 (4)	−0.1969 (2)	0.0984 (15)	
H14A	0.5518	0.2336	−0.2317	0.148*	
H14B	0.5115	0.3242	−0.1852	0.148*	
H14C	0.6245	0.2236	−0.1664	0.148*	
C15	1.2046 (4)	0.4562 (5)	−0.2066 (2)	0.0894 (14)	
H15A	1.1504	0.4138	−0.2370	0.107*	
C16	1.2464 (5)	0.5728 (5)	−0.2151 (2)	0.0938 (14)	
H16A	1.2203	0.6100	−0.2513	0.113*	
C17	1.3267 (4)	0.6362 (4)	−0.17070 (19)	0.0764 (12)	
H17A	1.3553	0.7160	−0.1765	0.092*	
C18	1.3637 (3)	0.5799 (4)	−0.11805 (17)	0.0645 (11)	
C19	1.3217 (4)	0.4635 (4)	−0.1092 (2)	0.0778 (12)	
H19A	1.3471	0.4261	−0.0730	0.093*	
C20	1.2418 (5)	0.4019 (4)	−0.1541 (3)	0.0903 (14)	
H20A	1.2130	0.3220	−0.1484	0.108*	
C21	1.3919 (4)	0.7121 (3)	−0.03685 (16)	0.0584 (10)	
C22	1.4700 (3)	0.8590 (3)	0.04237 (15)	0.0526 (9)	
C23	1.5852 (3)	0.9022 (4)	0.07914 (16)	0.0629 (10)	
H23A	1.6716	0.8719	0.0758	0.075*	
C24	1.5739 (4)	0.9892 (4)	0.12045 (17)	0.0661 (11)	
H24A	1.6528	1.0165	0.1451	0.079*	
C25	1.4473 (3)	1.0370 (3)	0.12614 (15)	0.0554 (9)	
C26	1.3334 (3)	0.9931 (4)	0.09065 (16)	0.0646 (10)	
H26A	1.2471	1.0230	0.0944	0.078*	
C27	1.3439 (3)	0.9049 (4)	0.04911 (17)	0.0688 (11)	
H27A	1.2645	0.8760	0.0253	0.083*	
C28	1.3265 (4)	1.1931 (4)	0.16771 (19)	0.0853 (13)	
H28A	1.3391	1.2493	0.2000	0.128*	
H28B	1.2499	1.1400	0.1699	0.128*	
H28C	1.3089	1.2386	0.1319	0.128*	

### Atomic displacement parameters (Å^2^)

**Table:**

	*U*^11^	*U*^22^	*U*^33^	*U*^12^	*U*^13^	*U*^23^
O1	0.0469 (13)	0.107 (2)	0.118 (2)	−0.0070 (13)	0.0267 (13)	−0.0378 (17)
O2	0.0419 (13)	0.0875 (19)	0.092 (2)	0.0005 (13)	0.0174 (12)	−0.0078 (15)
O3	0.085 (2)	0.085 (2)	0.0725 (19)	−0.0031 (17)	0.0130 (15)	0.0021 (17)
O4	0.0475 (14)	0.114 (2)	0.0847 (19)	−0.0007 (15)	0.0136 (14)	−0.0360 (18)
O5	0.0390 (14)	0.095 (2)	0.0863 (19)	−0.0060 (14)	0.0130 (12)	−0.0183 (16)
O6	0.0538 (15)	0.0753 (18)	0.0846 (19)	0.0041 (14)	0.0023 (13)	−0.0187 (16)
N1	0.0414 (16)	0.081 (2)	0.100 (2)	−0.0004 (17)	0.0213 (17)	−0.009 (2)
N2	0.0363 (14)	0.088 (2)	0.0609 (18)	0.0009 (16)	0.0069 (13)	−0.0087 (18)
C1	0.072 (3)	0.141 (4)	0.104 (4)	−0.021 (3)	0.009 (3)	−0.049 (4)
C2	0.098 (4)	0.176 (5)	0.076 (3)	0.010 (4)	−0.001 (3)	0.023 (3)
C3	0.083 (3)	0.107 (4)	0.101 (3)	0.033 (3)	0.012 (3)	0.033 (3)
C4	0.0358 (18)	0.075 (3)	0.077 (3)	−0.0032 (19)	0.0079 (18)	−0.004 (2)
C5	0.058 (2)	0.097 (3)	0.069 (3)	0.009 (2)	0.014 (2)	0.018 (2)
C6	0.074 (3)	0.083 (3)	0.119 (4)	−0.010 (3)	0.018 (3)	−0.008 (3)
C7	0.0469 (13)	0.107 (2)	0.118 (2)	−0.0070 (13)	0.0267 (13)	−0.0378 (17)
C8	0.046 (2)	0.059 (2)	0.087 (3)	0.0060 (19)	0.019 (2)	0.002 (2)
C9	0.058 (2)	0.079 (3)	0.073 (3)	−0.001 (2)	0.023 (2)	0.005 (2)
C10	0.057 (2)	0.081 (3)	0.072 (3)	−0.005 (2)	0.022 (2)	0.007 (2)
C11	0.059 (2)	0.057 (2)	0.073 (3)	0.008 (2)	0.011 (2)	0.006 (2)
C12	0.074 (3)	0.066 (3)	0.084 (3)	0.004 (2)	0.035 (2)	−0.002 (2)
C13	0.061 (2)	0.069 (3)	0.100 (3)	−0.003 (2)	0.039 (2)	−0.001 (3)
C14	0.091 (3)	0.102 (4)	0.103 (4)	−0.026 (3)	0.017 (3)	0.001 (3)
C15	0.061 (3)	0.111 (4)	0.096 (4)	−0.008 (3)	0.015 (3)	−0.037 (3)
C16	0.086 (3)	0.122 (4)	0.072 (3)	0.000 (3)	0.010 (3)	0.000 (3)
C17	0.071 (3)	0.081 (3)	0.075 (3)	−0.014 (2)	0.008 (2)	0.002 (3)
C18	0.041 (2)	0.088 (3)	0.065 (3)	0.002 (2)	0.0099 (19)	−0.014 (2)
C19	0.070 (3)	0.079 (3)	0.086 (3)	−0.004 (2)	0.016 (2)	0.009 (3)
C20	0.077 (3)	0.081 (3)	0.113 (4)	−0.015 (3)	0.018 (3)	−0.010 (3)
C21	0.046 (2)	0.066 (3)	0.063 (2)	0.002 (2)	0.0094 (19)	0.002 (2)
C22	0.0417 (19)	0.062 (2)	0.054 (2)	−0.0021 (18)	0.0065 (16)	0.007 (2)
C23	0.0374 (19)	0.081 (3)	0.069 (2)	−0.0005 (19)	0.0063 (17)	−0.001 (2)
C24	0.043 (2)	0.082 (3)	0.069 (3)	−0.003 (2)	−0.0026 (17)	−0.003 (2)
C25	0.041 (2)	0.064 (3)	0.059 (2)	0.0010 (18)	0.0039 (17)	0.003 (2)
C26	0.042 (2)	0.082 (3)	0.070 (3)	0.007 (2)	0.0102 (18)	−0.007 (2)
C27	0.0359 (19)	0.087 (3)	0.081 (3)	0.0007 (19)	0.0022 (18)	−0.012 (2)
C28	0.066 (3)	0.087 (3)	0.103 (3)	0.012 (2)	0.015 (2)	−0.021 (3)

### Geometric parameters (Å, °)

**Table:**

O1—C7	1.355 (5)	C10—H10A	0.9300
O1—C4	1.381 (4)	C11—C12	1.385 (5)
O2—C7	1.195 (4)	C12—C13	1.367 (5)
O3—C11	1.372 (4)	C12—H12A	0.9300
O3—C14	1.411 (4)	C13—H13A	0.9300
O4—C21	1.366 (4)	C14—H14A	0.9600
O4—C18	1.403 (4)	C14—H14B	0.9600
O5—C21	1.203 (4)	C14—H14C	0.9600
O6—C25	1.371 (4)	C15—C20	1.349 (6)
O6—C28	1.414 (4)	C15—C16	1.358 (6)
N1—C7	1.332 (5)	C15—H15A	0.9300
N1—C8	1.418 (5)	C16—C17	1.371 (6)
N1—H1A	0.8600	C16—H16A	0.9300
N2—C21	1.335 (4)	C17—C18	1.362 (5)
N2—C22	1.414 (4)	C17—H17A	0.9300
N2—H2B	0.8600	C18—C19	1.359 (5)
C1—C6	1.279 (6)	C19—C20	1.369 (6)
C1—C2	1.445 (4)	C19—H19A	0.9300
C1—H1B	0.9300	C20—H20A	0.9300
C2—C3	1.371 (7)	C22—C27	1.376 (5)
C2—H2A	0.9300	C22—C23	1.378 (5)
C3—C4	1.344 (5)	C23—C24	1.368 (5)
C3—H3A	0.9300	C23—H23A	0.9300
C4—C5	1.383 (5)	C24—C25	1.382 (5)
C5—C6	1.337 (6)	C24—H24A	0.9300
C5—H5A	0.9300	C25—C26	1.357 (5)
C6—H6A	0.9300	C26—C27	1.380 (5)
C8—C9	1.374 (5)	C26—H26A	0.9300
C8—C13	1.382 (5)	C27—H27A	0.9300
C9—C10	1.374 (5)	C28—H28A	0.9600
C9—H9A	0.9300	C28—H28B	0.9600
C10—C11	1.372 (5)	C28—H28C	0.9600
			
C7—O1—C4	120.3 (3)	O3—C14—H14B	109.5
C11—O3—C14	118.0 (3)	H14A—C14—H14B	109.5
C21—O4—C18	116.4 (3)	O3—C14—H14C	109.5
C25—O6—C28	117.2 (3)	H14A—C14—H14C	109.5
C7—N1—C8	125.9 (3)	H14B—C14—H14C	109.5
C7—N1—H1A	117.0	C20—C15—C16	120.0 (5)
C8—N1—H1A	117.0	C20—C15—H15A	120.0
C21—N2—C22	126.9 (3)	C16—C15—H15A	120.0
C21—N2—H2B	116.6	C15—C16—C17	120.6 (5)
C22—N2—H2B	116.6	C15—C16—H16A	119.7
C6—C1—C2	119.2 (5)	C17—C16—H16A	119.7
C6—C1—H1B	120.4	C18—C17—C16	118.7 (4)
C2—C1—H1B	120.4	C18—C17—H17A	120.6
C3—C2—C1	117.2 (5)	C16—C17—H17A	120.6
C3—C2—H2A	121.4	C19—C18—C17	121.0 (4)
C1—C2—H2A	121.4	C19—C18—O4	120.1 (4)
C4—C3—C2	120.8 (5)	C17—C18—O4	118.8 (4)
C4—C3—H3A	119.6	C18—C19—C20	119.3 (4)
C2—C3—H3A	119.6	C18—C19—H19A	120.3
C3—C4—O1	120.5 (4)	C20—C19—H19A	120.3
C3—C4—C5	119.9 (4)	C15—C20—C19	120.4 (5)
O1—C4—C5	119.3 (4)	C15—C20—H20A	119.8
C6—C5—C4	118.9 (4)	C19—C20—H20A	119.8
C6—C5—H5A	120.5	O5—C21—N2	128.2 (4)
C4—C5—H5A	120.5	O5—C21—O4	122.9 (3)
C1—C6—C5	123.9 (5)	N2—C21—O4	108.9 (3)
C1—C6—H6A	118.1	C27—C22—C23	117.9 (4)
C5—C6—H6A	118.1	C27—C22—N2	124.4 (3)
O2—C7—N1	127.8 (4)	C23—C22—N2	117.7 (3)
O2—C7—O1	123.0 (4)	C24—C23—C22	120.8 (3)
N1—C7—O1	109.2 (3)	C24—C23—H23A	119.6
C9—C8—C13	118.8 (4)	C22—C23—H23A	119.6
C9—C8—N1	122.3 (4)	C23—C24—C25	121.1 (3)
C13—C8—N1	118.8 (3)	C23—C24—H24A	119.5
C8—C9—C10	120.6 (4)	C25—C24—H24A	119.5
C8—C9—H9A	119.7	C26—C25—O6	125.2 (3)
C10—C9—H9A	119.7	C26—C25—C24	118.3 (4)
C11—C10—C9	120.6 (4)	O6—C25—C24	116.4 (3)
C11—C10—H10A	119.7	C25—C26—C27	121.0 (3)
C9—C10—H10A	119.7	C25—C26—H26A	119.5
O3—C11—C10	125.3 (4)	C27—C26—H26A	119.5
O3—C11—C12	115.7 (4)	C22—C27—C26	120.9 (3)
C10—C11—C12	118.9 (4)	C22—C27—H27A	119.5
C13—C12—C11	120.4 (4)	C26—C27—H27A	119.5
C13—C12—H12A	119.8	O6—C28—H28A	109.5
C11—C12—H12A	119.8	O6—C28—H28B	109.5
C12—C13—C8	120.6 (4)	H28A—C28—H28B	109.5
C12—C13—H13A	119.7	O6—C28—H28C	109.5
C8—C13—H13A	119.7	H28A—C28—H28C	109.5
O3—C14—H14A	109.5	H28B—C28—H28C	109.5
			
C6—C1—C2—C3	2.7 (8)	C20—C15—C16—C17	0.2 (7)
C1—C2—C3—C4	−0.7 (7)	C15—C16—C17—C18	0.0 (6)
C2—C3—C4—O1	173.6 (4)	C16—C17—C18—C19	−0.3 (6)
C2—C3—C4—C5	−0.9 (7)	C16—C17—C18—O4	176.2 (3)
C7—O1—C4—C3	106.2 (5)	C21—O4—C18—C19	−91.7 (4)
C7—O1—C4—C5	−79.3 (5)	C21—O4—C18—C17	91.7 (4)
C3—C4—C5—C6	0.5 (6)	C17—C18—C19—C20	0.4 (6)
O1—C4—C5—C6	−174.0 (3)	O4—C18—C19—C20	−176.0 (3)
C2—C1—C6—C5	−3.3 (7)	C16—C15—C20—C19	−0.1 (7)
C4—C5—C6—C1	1.7 (7)	C18—C19—C20—C15	−0.2 (6)
C8—N1—C7—O2	0.3 (8)	C22—N2—C21—O5	−4.3 (6)
C8—N1—C7—O1	179.2 (3)	C22—N2—C21—O4	175.8 (3)
C4—O1—C7—O2	−0.4 (7)	C18—O4—C21—O5	4.5 (6)
C4—O1—C7—N1	−179.4 (4)	C18—O4—C21—N2	−175.5 (3)
C7—N1—C8—C9	−34.6 (6)	C21—N2—C22—C27	−5.7 (6)
C7—N1—C8—C13	145.2 (4)	C21—N2—C22—C23	174.9 (3)
C13—C8—C9—C10	−0.8 (6)	C27—C22—C23—C24	−0.8 (5)
N1—C8—C9—C10	179.0 (3)	N2—C22—C23—C24	178.6 (3)
C8—C9—C10—C11	0.5 (6)	C22—C23—C24—C25	−0.7 (6)
C14—O3—C11—C10	−5.6 (5)	C28—O6—C25—C26	−18.3 (5)
C14—O3—C11—C12	171.3 (3)	C28—O6—C25—C24	165.1 (3)
C9—C10—C11—O3	177.2 (3)	C23—C24—C25—C26	1.7 (6)
C9—C10—C11—C12	0.3 (6)	C23—C24—C25—O6	178.6 (3)
O3—C11—C12—C13	−177.8 (3)	O6—C25—C26—C27	−177.9 (3)
C10—C11—C12—C13	−0.7 (6)	C24—C25—C26—C27	−1.3 (6)
C11—C12—C13—C8	0.3 (6)	C23—C22—C27—C26	1.2 (5)
C9—C8—C13—C12	0.4 (6)	N2—C22—C27—C26	−178.1 (3)
N1—C8—C13—C12	−179.3 (3)	C25—C26—C27—C22	−0.2 (6)

### Hydrogen-bond geometry (Å, °)

**Table:**

*D*—H···*A*	*D*—H	H···*A*	*D*···*A*	*D*—H···*A*
N1—H1A···O5	0.86	2.19	3.038 (4)	170
N2—H2B···O2^i^	0.86	2.22	3.062 (4)	166
C6—H6A···O5^ii^	0.93	2.58	3.435 (5)	153
C9—H9A···O2	0.93	2.52	2.967 (5)	110
C23—H23A···O2^i^	0.93	2.60	3.390 (4)	144
C27—H27A···O5	0.93	2.30	2.907 (5)	122

Symmetry codes: (i) *x*+1, *y*, *z*; (ii) −*x*+2, −*y*+2, −*z*.
